# Self-Referential Processing, Rumination, and Cortical Midline Structures in Major Depression

**DOI:** 10.3389/fnhum.2013.00666

**Published:** 2013-10-10

**Authors:** Ayna Baladi Nejad, Philippe Fossati, Cédric Lemogne

**Affiliations:** ^1^AP-HP, Service Universitaire de Psychiatrie de l’Adulte et du Sujet Âgé, Hôpitaux Universitaires Paris Ouest, Paris, France; ^2^USR 3246, CR-ICM, CNRS, Université Pierre et Marie Curie Paris-VI, Paris, France; ^3^AP-HP, Service de Psychiatrie d’Adultes, GH Pitié Salpêtrière, Paris, France; ^4^Faculté de Médecine, Sorbonne Paris Cité, Université Paris Descartes, Paris, France; ^5^U894, Centre Psychiatrie et Neurosciences, INSERM, Paris, France

**Keywords:** major depression, rumination, self-referential processing, neuroimaging, fMRI, medial prefrontal cortex, anterior cingulate cortex, default mode network

## Abstract

Major depression is associated with a bias toward negative emotional processing and increased self-focus, i.e., the process by which one engages in self-referential processing. The increased self-focus in depression is suggested to be of a persistent, repetitive and self-critical nature, and is conceptualized as ruminative brooding. The role of the medial prefrontal cortex in self-referential processing has been previously emphasized in acute major depression. There is increasing evidence that self-referential processing as well as the cortical midline structures play a major role in the development, course, and treatment response of major depressive disorder. However, the links between self-referential processing, rumination, and the cortical midline structures in depression are still poorly understood. Here, we reviewed brain imaging studies in depressed patients and healthy subjects that have examined these links. Self-referential processing in major depression seems associated with abnormally increased activity of the anterior cortical midline structures. Abnormal interactions between the lateralized task-positive network, and the midline cortical structures of the default mode network, as well as the emotional response network, may underlie the pervasiveness of ruminative brooding. Furthermore, targeting this maladaptive form of rumination and its underlying neural correlates may be key for effective treatment.

## Introduction

Major depressive disorder, or unipolar depression, is the greatest single contributor of all disease burden in the European Union (Wittchen et al., 2011). It is characterized by feelings of sadness and helplessness, anhedonia, lack of motivation, and social withdrawal. Early cognitive theories of depression posited that negative affect arises from the discrepancy between one’s internal representation of oneself, the perceived self, and one’s desired goals and attributes, the ideal self (Duval and Wicklund, 1972; Pyszczynski and Greenberg, 1987; Carver and Scheier, 1998; Higgins, 1999). There seems now to be a general consensus that an increased negative self-focus can lead to depression. Moreover, compared to healthy individuals, depressed patients have been found to attend more to negative stimuli and also to better recall negative stimuli than positive ones (Williams et al., 1996). For instance, in visual dot-probe tasks with emotional face stimuli, reaction times have been found faster for sad faces than neutral faces suggesting that depressive patients tend to direct attention to negatively valenced information (Gotlib et al., 2004). In depression, negative bias seems already apparent below the level of conscious awareness as evidenced by increased amygdala reactivity to masked sad faces (Victor et al., 2010). These biases could translate to an increased salience of negative life events that can reinforce the perceived shortcomings of the self, and if such things are dwelled upon, the individual is eventually drawn into a depressive episode (Teasdale, 1985).

The repetitive thinking and focus on negative mood states is referred to as rumination (Nolen-Hoeksema et al., 1993). There is a large body of observational and experimental evidence suggesting a reciprocally reinforcing relationship between rumination and negative affect (Mor and Winquist, 2002). Rumination tends to increase when negative emotions are up-regulated (Ray et al., 2005). In depressive patients, levels of rumination have been associated with the severity and duration of depressive episodes (Nolen-Hoeksema et al., 2008). Also, increased levels of rumination have been found to increase the risk of depressive relapse in remitted patients (Roberts et al., 1998).

Although content in ruminative thought is typically retrospective and self-depreciating (Watkins and Moulds, 2005), not all components of ruminative thinking are necessarily harmful (Treynor et al., 2003). The component referred to as “self-reflection” can be adaptive if the sense of agency in bettering one’s standing is retained. The maladaptive components of rumination are referred to as “brooding” and “depression-related,” and are associated with a greater negative bias (Joormann and Gotlib, 2006). Therapeutic interventions specifically targeting these maladaptive components of rumination, such as mindfulness-based cognitive therapy, have been found effective in preventing depressive relapse (Teasdale et al., 2000; Ma and Teasdale, 2004; Bondolfi et al., 2010).

Rumination is a form of self-referential processing, which is the process of relating information to the self. In neuroimaging, self-referential processing has been associated with the medial prefrontal cortex, anterior and posterior cingulate cortex, insula, temporal pole, hippocampus, and amygdala (Gusnard et al., 2001; Kelley et al., 2002; Fossati et al., 2003; Phan et al., 2004; Ochsner and Gross, 2005; Johnson et al., 2006; Schmitz and Johnson, 2006; van der Meer et al., 2010). In a meta-analysis of neuroimaging studies focused on self-referential processing, Northoff et al. (2006) found that commonly activated regions lie in dorsal and ventral areas of the medial prefrontal and anterior cingulate cortices, as well as the posterior cingulate cortex and precuneus. These regions have been termed cortical midline structures (Northoff and Bermpohl, 2004) and somewhat overlap with the intrinsic default mode network (Raichle et al., 2001; Spreng and Grady, 2010; Qin and Northoff, 2011). The default mode network is found in resting state functional imaging and as a deactivated network in functional imaging during cognitive task performance (Fox et al., 2005; Smith et al., 2009). When the brain is at rest, i.e., not engaged in externally driven cognitive processing, then self-referential processing is believed to predominate (Gusnard et al., 2001) and more activity in the default mode network is observed.

Here, we present a review of the neuroimaging literature (up until April 2013) investigating rumination or self-referential processing in major depressive disorder. These studies are discussed together with related literature that might help to elucidate the role of cortical midline structures in major depression and maladaptive self-focus. Firstly, we will review functional magnetic resonance imaging (fMRI) studies that have addressed the role of the cortical midline structures during self-referential processing in major depression. These studies are separated according to a discussion implicating cortical midline structures, and a discussion of the modulatory dynamics between the cortical midline structures, the amygdala, and the dorsolateral prefrontal cortex. Furthermore, we summarize results from resting state functional connectivity data that suggest abnormalities of the default mode network function and its relationship with the task-positive network in major depression, which could lead to maladaptive self-focus. Finally, we will consider evidence suggesting that antidepressant treatments target the neural bases of self-referential processing and rumination in their therapeutic effects.

### Self-referential tasks implicating cortical midline structures in major depression

Rumination has been found associated with the cortical midline regions, especially the more anterior portion. Kross et al. (2009) asked healthy subjects to adopt different thought processing strategies when recalling negative autobiographical memories during fMRI. The strategy which induced rumination, the repetitive and negatively toned style of self-referential processing, was found to increase neural activity in the subgenual anterior cingulate cortex and medial prefrontal cortex when compared to non-ruminative conditions. In addition, these same cortical midline structures have been implicated in the pathophysiology of depression. In a meta-analysis of 64 structural magnetic resonance imaging studies, the greatest brain volume reductions in depression were found in the anterior cingulate cortex and orbital frontal cortex (Koolschijn et al., 2009). Functional imaging with positron emission tomography (Videbech, 2000) and magnetic resonance imaging (Mayberg, 2002; Drevets et al., 2008) has also implicated cortical midline regions, the anterior portion in particular, in the pathophysiology of major depressive disorder.

More specific to rumination, functional imaging studies which experimentally probe self-referential processing have identified anterior cortical midline structures as key areas of dysfunction in depression (Lemogne et al., 2012). In one study (Grimm et al., 2009), positive and negative picture stimuli were presented to depressed patients and healthy comparison subjects under two conditions: passive viewing, and self-related judgment where subjects responded yes or no as to whether they could personally relate to the picture shown. Patients with depression compared to healthy control subjects were found hypoactive in cortical midline structures such as the dorsomedial prefrontal cortex and supragenual anterior cingulate cortex, as well as the dorsomedial thalamus and ventral striatum, during self-referential processing.

Instead of emotional picture stimuli, positive and negative personality trait words have also been presented to subjects under similar conditions where they were required to make a judgment as to whether or not the word applied to them. Using this task with a control condition requiring subjects to judge whether the word was a socially desirable trait or not, Lemogne et al. (2009) found that patients with unipolar depression recruited an extended portion of the anterior cortical midline structures. A part of the dorsomedial prefrontal cortex, which was not recruited by control subjects for self-referential processing, was found increased in activity during the self-judgment condition in depression patients. Interestingly, the dorsomedial prefrontal cortex appeared to remain hyperactive during self-referential processing in a small subsample who were re-scanned several weeks later (Lemogne et al., 2010). In a separate study using a similar task but with a control condition asking whether the trait word describes the current prime minister of the subjects’ country (Yoshimura et al., 2010), patients with major depressive disorder were found to exhibit hyperactivity in the medial prefrontal and rostral anterior cingulate cortices in the condition where they were asked to make a self-judgment for a negative trait word. These regions were hypoactive in comparison to healthy control levels in trials where positive trait words were presented.

Johnson et al. (2006) dissociated brain regions related to the type of content in self-referential processing in healthy control subjects, but, in a later study, found that these dissociations seemed not to apply for major depression patients. Self-referential thought related to hopes and aspirations tended to be associated with the anterior midline regions whereas self-referential thought related to duties and obligations was associated with the posterior cortical midline regions. In acutely depressed patients, the two kinds of self-related thoughts were not differentiated to the same extent as in control subjects (Johnson et al., 2009). This seemed to have been due to patients displaying hyperactivity during the control condition which led to less signal change in both self-related conditions. This pattern of activity was associated with a self-reported measure of rumination. With the same subjects they also investigated the distinction made by Watkins (2008) between two forms of self-focus: analytical self-focus – abstract thinking relating to the extended, narrative self – and experiential self-focus – concrete thinking concerned with one’s current state. The analytical type of self-focus, similar to ruminative brooding, has been suggested to evoke negative self-referential thoughts in major depression. Patients with depression tended to display hypoactivity in the medial prefrontal and anterior cingulate cortices during both analytical and experiential conditions as well as hyperactivity in these regions during the control condition. This activity pattern was again stronger for higher rumination scorers (Johnson et al., 2009).

In another study (Cooney et al., 2010), subjects were prompted to ruminate by being asked to think about statements relating directly to their sense of self. This condition was contrasted with when subjects were asked to think about abstract (e.g., the idea of team spirit) or concrete statements (e.g., seeing shampoo bottles on a store shelf). Patients with depression revealed to be hyperactive during rumination-induction in anterior and posterior cingulate cortex, as well as the dorsolateral prefrontal cortex, amygdala, parietal, temporal, and occipital lobes. In a study by Kessler et al. (2011), personally relevant material were gathered from intimate interviews and later presented to unmedicated depressed patients and healthy controls during fMRI. Compared to controls, patients were found to display greater activation of the medial prefrontal cortex but also, amongst other areas, the amygdala, raising the question of brain dynamics between cortical and limbic regions during self-referential processing in depression.

### The interplay of cortical and limbic regions during self-referential processing in depression

As well as cortical midline regions, the amygdala has been found abnormally recruited during self-referential processing in depressed patients (Cooney et al., 2010; Kessler et al., 2011). In a study solely investigating amygdala function, the bilateral amygdala response to self-referent emotional stimuli in remitted patients who underwent sad mood induction was found to predict the later increased recall of negative self-referential material (Ramel et al., 2007). This finding suggests an important role for the amygdala in the maintenance of depressive-related thought.

During a self-referential processing task where personally relevant negative, positive, and neutral words previously generated by the subject were presented during functional imaging, the amygdala exhibited a more sustained response to emotional stimuli in depressed patients and negatively correlated with rumination scores (Siegle et al., 2002). Along with the amygdala, the left hippocampus and dorsolateral prefrontal cortex also exhibited a more sustained response during self-referential processing. The dorsolateral prefrontal cortex is involved in the inhibition of limbic regions for the regulation of emotional response (Ochsner et al., 2012). However, considering that there are no direct anatomical connections between the amygdala and dorsolateral prefrontal cortex, the relationship is perhaps mediated by the medial prefrontal and anterior cingulate cortices. Animal studies examining anatomical connectivity of the amygdala have found, consistent over the different mammalian systems studied, prominent reciprocal connections to the medial prefrontal cortex with more elaborate amygdaloid connectivity to the forebrain in primate species (Price, 2003). Indeed, Siegle et al. (2007) in a follow-up study found that the variance of amygdala activity could be better explained by the activity of the anterior cingulate cortex than the dorsolateral prefrontal cortex. This study further found the anterior cingulate cortex hyperactive in response to negative stimuli and that the functional connectivity of the anterior cingulate cortex to the amygdala and to the dorsolateral prefrontal cortex was reduced in patients compared to controls.

Another study has found an increased connectivity between the amygdala and the medial prefrontal cortex in depression patients performing a self-referential word task (Yoshimura et al., 2010). However, in unmedicated depressed patients, functional connectivity between the amygdala and medial prefrontal cortex was observed decreased during rest and passive emotional picture-viewing (Anand et al., 2005). The conflicting findings of the latter two studies might be explained by a difference in the self-relatedness of the respective task stimuli. Self-referential processing might be modulating the amygdala and medial prefrontal connectivity, and differentially so in depressed patients compared to control subjects. Indeed, genetic liability for depression seems to influence the extent of the modulation derived from self-relatedness (Lemogne et al., 2011b). However, the different medication status of subject samples in these studies does not allow the ruling out of antidepressant drugs also exerting a modulatory influence on functional connectivity.

Findings from one study have also suggested that amygdala activity in depression was only disrupted in the negative self-referential condition, whereas abnormality in dorsolateral prefrontal and anterior cingulate cortices was found to be general and spanned across all conditions (Hooley et al., 2009). In this study, subjects were imaged while presented with recordings of their mothers either praising, or criticizing them, or discussing a neutral subject. Remitted, formerly depressed patients displayed hyperactivity in the amygdala during criticism but displayed hypoactivity in the dorsolateral prefrontal cortex and anterior cingulate cortex in all conditions compared to healthy individuals. This decreased prefrontal involvement in the task might indicate decreased cognitive control over emotional responsiveness in the remitted patients which might further explain the hyperactive amygdala response during the criticism condition.

In a self-referential task with personality trait words, Lemogne et al. (2009) found the medial prefrontal cortex displayed greater functional connectivity with the dorsal anterior cingulate cortex and the dorsolateral prefrontal cortex in patients with depression compared to controls. A study with healthy individuals (Wagner et al., 2012) found activity of the rostral anterior cingulate cortex to negatively co-vary with the activity in the dorsolateral prefrontal cortex. The activity in rostral anterior cingulate cortex was linked to negative self-referential processing and associated with depressive symptom severity. As will be later discussed, the rostral anterior cingulate cortex seems important for treatment response in depression (Pizzagalli, 2011; Fu et al., 2013) and perhaps this is due to its possible role as a hub between the limbic, the self-referential, and the cognitive control networks.

### Resting state inter-regional dynamics of cortical midline regions in major depression

Aberrant rest-stimuli interactions has been suggested as a core dysfunction in depression which could underlie many of the symptoms including increased negative self-focus (Northoff et al., 2011). One study comparing whole-brain functional brain connectivity to a default mode network node in depressive patients and healthy control subjects, only found abnormal connectivity during the rest epochs and not during epochs of emotional word recall (Berman et al., 2011). This is also in line with the previously mentioned studies of self-referential processing suggesting that depression patients exhibit abnormal activity already during control conditions (Hooley et al., 2009; Johnson et al., 2009).

However, activity within rest epochs have been found to be dependent on the cognitive processing of the preceding condition. For example, task difficulty in working memory conditions affects subsequent rest periods (Pyka et al., 2009, 2012), but perhaps more relevant here, the self-relatedness of stimuli has also been found to affect subsequent rest periods (Schneider et al., 2008). If depressive patients differentially process emotional stimuli then it would follow that the rest epochs would also differ from comparison subjects. Furthermore, depressed patients might also have a more sustained neural response to emotional/self-referential stimuli, as Siegle et al. (2002) suggest, which could spill over into rest periods.

Most of functional brain imaging in present times use magnetic resonance imaging to pick up changes in the blood oxygenation level-dependent (BOLD) signal, a proxy for neural activity. Task activation studies traditionally use a mass-univariate general linear modeling approach to contrast implicit resting baselines to task-related activity peaks, i.e., relative signal changes are measured. With resting state activity there are no experimental parameters to which to model BOLD activity. Activity fluctuations are seen in relation to other regions in the brain with the assumption that regions with similar brain activity patterns are communicating with each other, i.e., the degree of functional connectivity between regions is measured. The resting state connectivity of parcellated brain regions, as assessed by correlation coefficients, within the default mode network, as well as the affective network, visual cortex, and cerebellum, were found to be highly discriminative of patients with depression from healthy control subjects using a multivoxel pattern classifier (Zeng et al., 2012). Differences in functional connectivity during resting state between patient and control groups could suggest that aberrant activity in depression is intrinsic and not only related to self-referential processing. Alternatively, connectivity differences could be reflective of qualitatively different thought content during rest in depressed compared to healthy individuals. Furthermore, resting state connectivity might be affected by antidepressant drugs and/or depressive episode duration. Increased resting state functional connectivity of the precuneus and the thalamus to the rest of the default mode network was found in depressed patients, and this increase also correlated with the duration of depressive episode (Greicius et al., 2007). Default mode network dysconnectivity was, however, already apparent in treatment-naïve patients with first-episode depression (Zhu et al., 2012). These latter two studies were similar in that they both used independent component analysis to investigate resting state activity but they differed in the direction of the dysconnectivity finding in patients. Whereas Greicius et al. found increased default mode network connectivity of posterior regions, Zhu et al. found posterior default mode network connectivity to be decreased in patients compared to healthy control subjects.

The Zhu et al. (2012) study also found default mode network connectivity in the anterior regions, ventral medial prefrontal, and anterior cingulate cortices, to be increased in depression compared to healthy control levels and this was also positively correlated with rumination scores. In another resting state study of depressive patients (Sheline et al., 2010), the dorsomedial prefrontal cortex, was found to exhibit an increased connectivity to seed regions representative of the cognitive, default mode, and affective networks suggesting that the anterior cortical midline might mediate the dysfunction of these networks which has been previously reported in depression. In a seed-based functional connectivity analysis of anterior cingulate resting state activity in young depressive patients, Davey et al. (2012) also found that the dorsomedial prefrontal cortex displayed increased connectivity, which the authors suggest could be reflective of the increased self-referential processing in patients.

A dysfunctional dorsolateral prefrontal cortex has been implicated as perhaps a precursor to the hyperactive midline cortical processing found in depression (Marchetti et al., 2012). The resting state functional connectivity between the dorsolateral prefrontal cortex and anterior cingulate cortex was found increased in depressive patients, which might suggest altered cognitive regulation processes that can lead to the negative self-focus in depression (Davey et al., 2012). In support of a cognitive disinhibition leading to increased rumination, many task activation studies which have probed self-referential processes in depression patients have also found anomalies in the dorsolateral prefrontal cortex, a key node of the task-positive, cognitive control network (Siegle et al., 2007; Lemogne et al., 2009; Cooney et al., 2010; Yoshimura et al., 2010; Kessler et al., 2011). Additionally, one study found that the integrity of superior longitudinal fasciculus (the frontoparietal fiber tract) was compromised in major depression patients and the degree of white matter integrity was negatively correlated with rumination scores (Zuo et al., 2012).

Cognitive control, or the lack of it, seems to also be an important factor in determining the degree of rumination a healthy individual normally engages in. In a task activation study with healthy subjects performing an emotional go/no-go task (Vanderhasselt et al., 2011), those who were reported as high ruminative brooders exhibited increased dorsolateral prefrontal cortical activation compared to low ruminators. This might suggest greater cognitive recruitment to overcome the emotionality of the stimuli which tends to be more salient in those prone to rumination. Another study (Kuhn et al., 2012) found that rumination was associated with gray matter volume reductions in the anterior cingulate cortex and inferior frontal gyrus which also overlapped with regions whose functional connectivity during rest was associated with rumination. The authors suggest that the associations to these areas, which have roles in cognitive inhibition, are indicative of a deficit in the suppression of ruminative thought.

The dynamics between the cognitive network and the default mode networks can also be seen from a bottom-up perspective where increased maladaptive self-focus and thereby hyperactive cortical midline regions interfere with normal cognitive function. One resting state study using a metric of network dominance looked at the relationship between rumination and resting state networks in patients with depression (Hamilton et al., 2011). The authors found that the activity of the default mode network was more dominant than the cognitive, task-positive network in the resting state profiles of depression patients compared to healthy control subjects. The degree of dominance of the default mode network was also positively correlated with scores on maladaptive rumination and negatively correlated with the more adaptive, “self-reflective” rumination. Hence, this default mode network dominance could explain the invasiveness of rumination in depression.

### Self-referential processing and cortical midline structures as treatment targets

The cortical midline structures have been found important for treatment response in depression. A strengthening of the connectivity between the anterior cingulate cortex and amygdala has been associated with symptom remission after antidepressant treatment in patients (Chen et al., 2008). Deep brain stimulation of the subgenual anterior cingulate cortex ameliorates symptoms in treatment-resistant depression (Mayberg et al., 2005). The same region’s pre-treatment level of response to negative words has been found predictive of response to cognitive therapy in depression (Siegle et al., 2012). Also, the resting state activity of the rostral anterior cingulate cortex has been found predictive of pharmacological treatment response in depression whereas posterior midline areas predicted treatment non-response according to a meta-analysis of longitudinal studies in depression (Pizzagalli, 2011).

A self-referential task was used to study the acute effect of citalopram, a selective serotonin reuptake inhibitor, in high neuroticism scorers who are thought to be at increased risk for depression. After administration of citalopram, high neuroticism scorers displayed decreased activity in the medial prefrontal and rostral anterior cingulate cortices in response to negative self-referential processing compared to high neuroticism scorers who had received a placebo (Di Simplicio et al., 2012). This study suggests that one mechanism by which antidepressants exert their action might be through the inhibition of negative self-referential processing in depression. A similar finding was reported in a study of remitted depression patients where neural response to negative emotional faces were attenuated in depression patients in remission compared to controls (Thomas et al., 2011). This study found that the neural response to negative stimuli was positively correlated with rumination scores which suggest that decreased neural activity is protective against rumination and hence depressive symptoms. In another study, which also found a positive correlation between negative stimuli neural response and rumination scores in remitted patients, the reactivity of the medial prefrontal cortex was further found to predict relapse status 18 months later (Farb et al., 2011). Similarly suggesting attenuated response to negative stimuli as therapeutic, healthy subjects were found to display reduced negative bias and reduced neural response to negative stimuli following a mindfulness task (Paul et al., 2013).

Another possible mechanism of action in antidepressants might be through increasing salience and neural processing of positive stimuli (Harmer et al., 2003, 2004; Miskowiak et al., 2007; Norbury et al., 2008). Yoshimura et al. (2013) found that, after cognitive behavioral therapy in depression patients, there was an increased neural response in the medial prefrontal and anterior cingulate cortices to positive, self-referential stimuli together with a decreased neural response in the same regions to negative stimuli. However, in a longitudinal investigation using a similar self-referential processing task as the latter, the medial prefrontal cortical hyperactivity found in a small sample of depressed patients did not change over weeks of antidepressant treatment (Lemogne et al., 2010). Additionally, a study with healthy subjects treated 3 weeks with escitalopram who performed a similar self-referential processing task, found the greatest treatment effect to lie in the precuneus and posterior cingulate cortex (Matthews et al., 2010). These discrepancies might result from the different therapeutic interventions, for example, acute versus prolonged antidepressant drug treatment, or antidepressant drug treatment versus cognitive behavior therapy.

## Discussion

**Figure 1:**
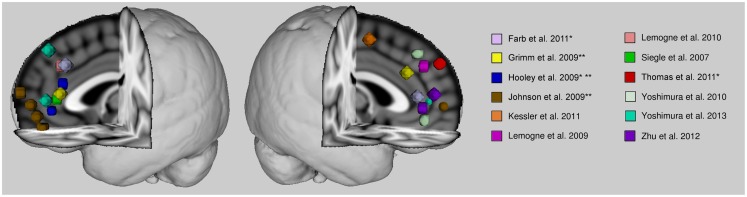
**Anterior cortical midline findings of previous studies reporting self-related abnormalities in depression**. Spheres are centered on peak voxel values of regions whose activities are reported significantly different in major depressive patients from healthy control subjects during self-referential processing, or during another paradigm (e.g., rest or emotional face processing) where activity was associated with rumination scores. Talairach coordinates were converted into MNI space with BrainMap toolbox (brainmap.org). Activity is greater in patients than control subjects unless otherwise indicated. *With a remitted patient sample. **Hypoactivity in patients.

The anterior cortical midline structures, namely, medial prefrontal cortex and anterior cingulate cortex, seem implicated in the abnormal self-referential related activity of patients with major depression. This abnormality tends toward an increased level of activity. Studies finding decreased activity of these regions during self-referential processing in patients had also found increased activity in the comparison conditions (Hooley et al., 2009; Johnson et al., 2009), and therefore the apparent hypoactivity could be relative due to the statistical contrast. Overall, studies suggest that there is an aberrantly increased level of anterior cortical midline activity, if not during self-referential processing, then basally. There seems to be some discrepancy in the findings as to the specific localization within the anterior midline cortex of this dysfunction (see Figure 1). The discrepancy might be due to the broad range of tasks employed in the literature as well as the methods employed to analyze the data. Many studies opt for a region-of-analysis approach varying in their method of region selection. There is variability even in whole-brain analyses when it comes to statistical thresholding which might affect the reliability of some results. Also, some findings are based on small samples and this might also contribute to some inconsistencies in the literature.

Some studies have identified an abnormally increased responsiveness of the anterior cortical midline regions during specifically negative self-referential conditions (Siegle et al., 2007; Yoshimura et al., 2010, 2013; Wagner et al., 2012). However, studies have also reported a behavioral difference in conditions where a judgment needs to be made as to whether the positive or negative stimuli is something the participant can personally relate to (Siegle et al., 2007; Grimm et al., 2009; Lemogne et al., 2009; Yoshimura et al., 2010). Patients with depression tend to personally relate to negative stimuli more often than control subjects, and also personally relate to positive stimuli less often than control subjects. No study to date has looked at whether the processing of self-related negative stimuli is abnormally recruiting the midline cortical region, or whether the abnormal activity reported in this region is a proportional, “normal” response in light of the behavioral differences between groups, where patients seem to be relating negative stimuli more often to the self than control subjects. If the latter is true then the activity in the cortical midline region might not be pathological *per se* but just reflect a normal neural reaction to negative self-related material. The accounting of behavioral differences will be an interesting avenue for future studies to explore.

There is growing evidence for dysfunctional interactions between regions related to emotion, self-referential, and higher cognitive processing. In animal neuroanatomical studies, prominent reciprocal connections have been identified between the medial prefrontal and anterior cingulate cortices and the amygdala as well as with the thalamus, ventral striatum, and hypothalamus (Ongur and Price, 2000). The interactions between these regions play a significant part in the regulation of emotional and visceral response, and are therefore implicated in the pathophysiology of mood disorders (Price and Drevets, 2010, 2012). In the self-referential task-related imaging literature, the anterior cortical midline structures also seem to be repeatedly identified as mediators between the amygdala, an area associated with emotional response, and the dorsolateral prefrontal cortex, an area associated with cognitive control and emotion regulation. The functional connectivity of the anterior cortical midline structures to the amygdala and dorsolateral prefrontal cortex has been found disrupted in patients with major depression (Anand et al., 2005; Siegle et al., 2007; Lemogne et al., 2009; Yoshimura et al., 2010; Wagner et al., 2012). The resting state literature in parallel offers support to the idea that disrupted cognitive control leads to intrusion of ruminative thought, and ruminative thought has been further associated with increased connectivity and activity of the default mode network (Sheline et al., 2010; Hamilton et al., 2011; Davey et al., 2012; Marchetti et al., 2012). Rumination could stem from a top-down failure, where lack of inhibition from dorsolateral prefrontal regions to the anterior cingulate cortex allows free reign of ruminative thoughts. Or the failure could be a bottom-up process where overactive limbic regions tag negative emotionality and salience to experiences which lead to increased rumination and thereby to an interference with normal higher cognitive function and control. Although functional connectivity analyses have revealed dysconnectivity in major depression, more causal connectivity analyses could better identify the nature of this modulatory impairment.

The relationship between self-referential processing and abnormal activity in the cortical midline areas has not been replicated in medication-naïve patients to verify that the abnormality does not stem from antidepressant effects. One small pilot study examined whether these patterns of activation were stable over sustained antidepressant treatment (Lemogne et al., 2010). Additionally, one study examined the neural correlates of self-referential processing among a drug-naïve, at-risk for depression sample (Lemogne et al., 2011a). These latter two studies provided some evidence for trait-like hyperactivity in the dorsomedial prefrontal cortex. However, Yoshimura et al. (2013) recently found that this same region might be modulated by cognitive behavior therapy. Also, a study on at-risk, healthy subjects (high neuroticism scorers) found that hyperactive ventromedial prefrontal cortex response decreased after 7 days of citalopram treatment (Di Simplicio et al., 2012). Therapeutic effects of antidepressant treatment seem to be related to either a decreased neural response to negative self-referential stimuli or an increased response to positive self-referential stimuli. However, therapeutic actions of antidepressants on self-referential related activity have been more often than not studied in healthy individuals. Only a few longitudinal imaging studies have been conducted with patient samples. Also considering the delayed therapeutic response of antidepressants, a longer intervention period would be desirable in future studies in order to relate the therapeutic changes in self- and depressive-related symptoms with neural activity change.

## Conclusion

Although there may be some discrepancies between studies, there nonetheless seems to be a general convergence on the anterior cortical midline structures as playing an important role in maladaptive rumination in major depression. Evidence in support of this is derived from task-related as well as resting state data. Anterior cortical midline structures have been found to display abnormally increased activity usually in relation to negative self-focus. There is also evidence that activity within the cortical midline regions is associated with behavioral measures of rumination. The pervasiveness of ruminative thought in depression might be driven by a reduced top-down inhibition of the cortical midline and limbic regions, thereby allowing for the predominance of negatively charged and self-focused thought. In line with this, the dorsolateral prefrontal cortex has been found dysfunctional during self-referential processing and its dynamics with the emotional and self-referential associated brain regions seem also disrupted in depression. However, whether these disruptions arise from a top-down or a bottom-up mechanism is not yet clear. Moreover, too few longitudinal studies have been conducted in order to determine to what extent these neural dysfunctions are a precursor or a product of the depressive state, and to what extent does medication influence brain dynamics.

**Definition Box**

*Self-referential processing* is the cognitive process of relating information, often from the external world, to the self.

*Self-focus* refers to attention directed inwardly, to the self, as opposed to the external world.

*Rumination* is repetitive and distressful form of thinking that can be symptomatic of depression. Adaptive forms of rumination, however, have been identified where the content can be positive and can lead to problem resolution.

## Conflict of Interest Statement

Ayna Baladi Nejad reports no potential conflicts of interest. Philippe Fossati has received grants from Servier and honoraria from Lundbeck and Servier. Cédric Lemogne has accepted paid speaking engagements in industry-sponsored symposia from Astra Zeneca, Lundbeck, Pierre Fabre, Pfizer, and Servier.
